# Understanding Inequalities Shaping Engagement With Cultural and Green Assets to Improve Mental Health for Underserved Communities: A Qualitative Study

**DOI:** 10.1111/hex.70912

**Published:** 2026-10-08

**Authors:** Francesca Taylor, Tushna Vandrevala, Silke Zschomler, Meg Jensen, Ima Miah, Catherine Dawkins, Eneida Capaldi, Anna Bosher, Louise Bloom, Maria Chatzichristodoulou

**Affiliations:** ^1^ School of Nursing, Allied and Public Health Kingston University Kingston upon Thames London UK; ^2^ University of the Arts London London UK; ^3^ School of Creative and Cultural Industries Kingston University Kingston upon Thames London UK; ^4^ Asian Resource Centre of Croydon Croydon UK; ^5^ Connect: North Korea New Malden Surrey UK; ^6^ Kingston Voluntary Action Kingston upon Thames London UK; ^7^ The Reach Foundation Feltham Middlesex UK; ^8^ Refugee Action Kingston Kingston upon Thames London UK

**Keywords:** communities, community assets, ethnic minorities, health inequalities, mental health, migrants, refugees

## Abstract

**Introduction:**

Underserved communities including ethnic minorities, refugees and migrants, experience higher mental health needs than the general population, yet engage less with cultural and green community assets (CGCAs) that could support wellbeing. While access barriers are known, less is understood about the cultural meanings and processes underpinning inequalities shaping engagement. This study examined the cultural drivers and mechanisms of these inequalities and how they might be addressed to improve mental health.

**Methods:**

A multi‐site qualitative study in England with community voice champions (CVCs) recruited through five underserved community organisations. A lived experience storytelling methodology with thematic analysis was used, informed by a theoretical model of cultural processes and causal pathways to inequality. Twelve workshops were held, following which CVCs collected community stories.

**Results:**

Twenty‐seven CVCs participated and collected 147 stories. Symbolic, place‐based and material inequalities emerged across three themes: cultural influences on mental health; unknown spaces and unfriendly environments; and creating spaces of safety and belonging. Even when CGCAs were available and known, racial stigmatisation and cultural dissonance reinforced exclusion. Community‐led initiatives, facilitation and peer support helped make CGCAs safer and more welcoming.

**Conclusions:**

Engagement with CGCAs is not only about access, but a negotiated process shaped by intertwined inequalities. Nevertheless, these inequalities are not fixed. Community‐led activities grounded in trust and collective action can transform exclusionary environments into inclusive ones to support mental health.

**Patient or Public Contribution:**

Community organisation leads contributed to study design, participant recruitment and support, data interpretation, and are co‐authors. CVCs participated in workshops, collected and interpreted data, and validated findings.

## 1 Introduction

Mental ill‐health is a significant and growing public health concern globally [1, 2]. Rising prevalence rates have been reported both before and, more markedly, since the COVID‐19 global pandemic [3, 4], exacerbated by increasing workforce shortages in mental health services [4, 5]. However, the burden of mental illness is not equally shared across populations. Underserved communities including ethnic minorities, refugees, migrants, and asylum seekers, experience disproportionately high mental health needs relative to the general population [6, 7].

Mental health problems can persist for many years after migration and settling in a high‐income country [7, 8], influenced by pre‐ and post‐migration factors including trauma [9, 10], acculturation and assimilation processes [11], and insecure status [12]. Despite elevated mental health needs, underserved communities remain less likely to access formal mental healthcare services [13, 14]. Barriers include limited local services, and psychosocial factors such as perceived racism, language barriers and culturally incongruent care models [13, 14, 15].

There is growing recognition of the need to complement efforts to improve access to statutory mental health services with investment in community‐based, non‐clinical resources, such as nature, culture, and creative therapy to address persistent mental health inequalities [13, 16]. The World Health Organization (WHO) advocates extending mental health solutions beyond macro‐level systems to community‐led initiatives that incorporate recovery‐oriented practices responsive to the needs of underserved communities [17]. This approach emphasises collaboration between the health and arts sectors to enhance cultural capital and ensure equitable access [18]. The WHO and the Organisation for Economic Co‐operation and Development also recognise urban green spaces as vital community assets for promoting mental wellbeing [19, 20].

In England, policy promotes the use of cultural and green community assets (CGCAs) to support mental and physical health and reduce health inequalities. CGCAs include allotments, libraries, waterways, parks, museums, galleries, commons, art galleries, faith places and public gardens [21, 22]. National strategies emphasise the importance of connecting communities with nature to ensure equitable access to its health benefits [23, 24]. Several policy initiatives also highlight the role of arts and culture in improving mental health, including through social prescribing programmes [25, 26, 27].

There is substantive evidence linking engagement with CGCAs to positive impacts on mental health [28, 29], influenced by psychosocial, social and behavioural factors [30, 31]. For underserved communities these include respite from daily stress [32, 33]; sensory and memory‐based connections that foster belonging [32, 33, 34, 35]; opportunities for cultural and social interaction [36]; and spiritual succour [37]. Nevertheless, CGCAs remain underused by underserved communities [38, 39]. One contributory factor is that these communities often live in more deprived urban areas with limited access to green spaces [37, 40]. Barriers to access are well documented, in particular, travel difficulties, safety concerns, inadequate welfare facilities, language, and lack of cultural relevance and connection [41, 42]. Existing evidence, however, often overlooks the structural and causal factors underpinning inequalities influencing engagement with CGCAs. Deeper insight is needed into the cultural meanings and processes that shape how underserved communities engage with these assets [43], grounded in participatory understandings ‘from within’ communities [44].

The aim of our study was to address this evidence gap by examining the cultural drivers and mechanisms of inequalities that hinder underserved communities' engagement with CGCAs, and how these inequalities might be addressed to improve mental health. It forms part of a larger mixed‐methods project co‐designing and evaluating community‐led approaches to reduce mental health inequities through CGCA engagement [45].

### 1.1 Theoretical Framework

We used the theoretical framework of Lamont et al. to guide the study data analysis and interpretation [46]. This evidence‐based framework posits that community‐level cultural processes shape social structures which directly or indirectly produce social inequalities that link to health inequalities. It delineates three interrelated dimensions of inequality: material (e.g., housing, food, transport); place‐based (e.g., neighbourhood effects, local resources); and symbolic (e.g., knowledge, boundaries, exclusion).

Within this framework, two core cultural processes influencing meaning‐making operate at the community level, ‘identification’ (e.g., racialisation and stigmatisation) and ‘rationalisation’ (e.g., standardisation imposing habitual uniformities, and evaluation defining who or what is valued) [46]. We applied this framework as an analytical lens to understand shared cultural meanings embedded in the lived experiences of underserved communities and how these shape inequalities in engagement with CGCAs.

## 2 Methods

### 2.1 Design

A qualitative multi‐site study design was used in which data were collected using lived experience storytelling, incorporating snapshot story exercises, expressive life writing, and intentional listening [47, 48]. The strength of this methodology is that it enables identification and understanding of nuanced and non‐explicit cultural and ethnic influences, especially among vulnerable communities [48].

### 2.2 Setting

The study was conducted across five community organisations in Southwest London. Although the region includes some of the least deprived London boroughs, there are pockets of deprivation where underserved communities predominantly reside. Southwest London is among the most diverse populations in England, with around a third (35.5%) of the region's residents belonging to Black (12.5%), Asian (11%), mixed or multiple ethnic (6.7%), or other ethnic (5.3%) groups as compared with just under a fifth (19%) in England overall [49]. These communities experience relative social deprivation with higher rates of homelessness and temporary accommodation such as refugees and asylum seekers living in ‘asylum hotels’ [50]. The five sites provided variation in size, work focus and the underserved communities they supported (Table 1).

**Table 1:** Study site characteristics.

Study site	Characteristics
1	Umbrella organisation helping create healthier underserved communities through the support of a large number of charities and community organisations, of which over 20 are for ethnic minority groups, at all stages of development.
2	Single organisation supporting the health and wellbeing of Asian communities through community engagement and volunteer initiatives.
3	Single organisation supporting a multicultural population of children, young people and families including the local Asian community, through building and empowering community relationships and partnerships.
4	Single organisation supporting refugees and asylum seekers from Central America, Syria and Ukraine to integrate and rebuild their lives.
5	Single organisation supporting the mental and physical health of North Korean refugees.

### 2.3 Participants and Recruitment

The five community organisation study sites participated in response to an invitation to the senior lead, from the study principal investigators, to engage as community co‐investigators. The overall project was co‐designed with the community co‐investigators and they each recruited up to seven volunteer community voice champions (CVCs) to participate throughout the project. These volunteer posts were recruited through pre‐existing user groups and networks within each community organisation site, using advertising flyers and word‐of‐mouth.

Eligibility criteria for CVCs included being aged 18 years or older (16 years or older for one organisation working with younger adults), ability to give consent, and willingness to promote the project in their community, bring relevant information back to the research team, collect lived experience stories from members of their community, and undertake training. Those expressing interest in participation were sent information on the project objectives and what participation would involve by the relevant community co‐investigator who subsequently selected the CVCs. Selection was based on the eligibility criteria. From a total of 37 potential CVCs that applied, 10 were not selected to take part.

Following recruitment to the overall project, each CVC was sent an invitation by the research team to participate in this study, together with an information sheet outlining the study purpose and what participation would involve, and consent form. After providing additional oral explanation, written consent was taken from each CVC by a study researcher prior to the study start.

CVCs were re‐imbursed for their time, carer support and travel. They received a joint online study induction and mental health awareness training. They also received training on how to invite members of their own communities to share through conversation with them, anonymous lived experience stories on engaging with CGCAs and the wellbeing benefits. The study team provided ongoing support, maintaining safe, supportive spaces and offering one‐to‐one mental health support from an accredited university mental health nurse. Translation was available and used by two participants.

### 2.4 Data Collection

Twelve lived experience storytelling workshops were undertaken, facilitated by a research team member (MJ) with expertise in lived experience storytelling methodologies. Each CVC participated in a cycle of four workshops: two in person at their respective community organisations, and two online with all CVCs.

Participants were encouraged to share their stories through speaking, drawing or writing. The facilitator used semi‐structured storytelling prompts (Supporting Information S1: Supplement A) and techniques of checking interpretation and understanding. Participants who produced a drawing were asked to provide a verbal explanation of their drawing. The workshops lasted 55–142 min (median 112). With participants' consent, the sessions were audio‐recorded and transcribed, and contemporaneous notes taken by two study researchers (F.T., S.Z.).

During the third workshop CVCs received training by the facilitator on how to collect anonymous lived experience stories from community members on engaging with CGCAs and the wellbeing benefits. Before the fourth workshop they collected these stories from their own community members through informal conversations in community public spaces, such as community centres, cafes and places of worship, on as many occasions they chose over 3 weeks. CVCs were asked to explain to community members the project purpose and that all stories would remain anonymous. Storytelling prompts developed by the study research team were used to guide discussions, including questions about experiences of using a cultural or green space in their community area. CVCs were encouraged to take written notes and create drawings to capture the stories, and to check their understanding by repeating key parts of each story back to the to the storyteller. These stories were subsequently shared by CVCs with the study researchers. At the end of the fourth workshop, CVCs were asked by the facilitator to write a letter to their community, reflecting on insights from their storytelling conversations. Data were collected between September 2024 and February 2025.

### 2.5 Data Analysis

Two data sets were analysed: (1) CVC workshop transcripts, including verbal explanations of participant‐produced drawings, and CVC letters; and (2) community member stories collected by CVC participants, and written explanations of drawings they made about community member stories. The datasets were first analysed separately. Two study researchers (F.T., S.Z.)—an applied health services researcher with a social anthropology background, and a multi‐disciplinary social scientist—who between them attended all workshops, read and re‐read the first three workshop transcripts and 25 stories to identify preliminary codes and categories. All data were then coded and thematically analysed using inductive (data driven) and deductive (theory and literature‐informed) approaches [51]. A separate coding framework and coding index were developed for each dataset and used to structure verbatim responses onto Excel spreadsheets. Codes and themes were iteratively refined as further data from the datasets were incorporated, with some codes combined or removed. Emerging codes and themes were reviewed with a third study researcher (TV), a health psychologist specialising in health inequalities. The process was collaborative and reflexive, prioritising interpretive understanding over consensus with the aim of developing themes that offered ‘analytic outputs’ interpretive of the data [52].

The community co‐investigators and CVCs were invited to a face‐to‐face collaborative event with the study team in May 2025, for collective sense‐checking and refinement of emerging themes and subthemes. A further stage of deductive consideration and interpretation of the refined themes was undertaken by two researchers (T.V., F.T.), enabling deeper understanding of how the identified cultural processes and dimensions of inequality shaped engagement with CGCAs. Final themes were confirmed collaboratively with all study stakeholders.

The paper conforms to the standards for reporting qualitative research (SRQR) [53].

### 2.6 Ethics

Ethical approval for the study was received from the Ethics Committee of University of the Arts London (H&SCS‐CREC7075; 30 August 2024) and from the Ethics Committee of Kingston University (KUREOS 3563; 9 September 2024). Participation in the study was voluntary, with informed consent obtained from all participants before involvement.

## 3 Results

Twenty‐seven CVCs participated across the five study sites: Site 1 (seven CVCs), Site 2 (six), Site 3 (six), Site 4 (four), and Site 5 (four). Four CVCs withdrew during the study due to competing life pressures. Demographic data were not systematically collected by study researchers from CVCs because of the sensitivity surrounding their protected characteristics. Information volunteered by individual CVCs during participation in the lived experience storytelling workshops showed that participants represented a diverse range of ethnic and cultural backgrounds, including Indian, Afghan, North Korean, Arab, Tamil, Filipino, Pakistani, Caribbean, Somali, Uganda, and Kenyan. Seventeen participants described themselves as female and ten as male.

Eighteen CVCs collected 147 community member stories from 147 different individuals. Similarly, demographic data were not collected by CVCs from community members who provided lived experience stories because of sensitivity surrounding their protected characteristics. Fourteen CVCs wrote letters to their community.

Data analysis resulted in the identification of three overarching themes related to inequalities that influence community engagement with CGCAs. Themes and subthemes are summarised in Table 2.

**Table 2:** Summary of overarching themes and sub‐themes.

Overarching themes	Subthemes	CGCA engagement inequalities^a^
1. Cultural Influences on Mental Health	Gaps in understandings of mental health	Symbolic inequality
	Displacement, exclusion and desire to belong	Place‐based inequality
	Taboo, shame and silence	Symbolic inequality
2. Unknown Spaces and Unfriendly Environments	Limited awareness of CGCAs	Symbolic inequality
	Insecure and unsafe about accessing spaces	Material inequality/Place‐based inequality
3. Creating Spaces of Safety and Belonging	Spaces as sanctuary	Place‐based inequality
	Cultural dissonance and exclusion	Symbolic inequality
	Navigating unknown rules and institutional mistrust	Symbolic inequality
	Community makes inclusive, welcoming and safe spaces	Symbolic inequality
	Community facilitation and peer support	Place‐based inequality

^a^ Adapted from Lamont et al. 2014 [46].

### 3.1 Theme One: Cultural Influences on Mental Health

Participants often mentioned limited understanding of mental health, yet their accounts showed how loss, exclusion and racism intertwined to negatively impact wellbeing. A sense of belonging emerged as central to wellbeing, yet exclusionary practices and limited cultural awareness in host communities hindered integration and recovery. Cultural norms linked to privacy and reputation also discouraged discussing distress, restricting access to support.

#### 3.1.1 Gaps in Understandings of Mental Health

Participants frequently struggled with understanding mental health terminology, such language being uncommon within their cultural discourse. They tended to use words descriptive of feelings, including ‘panic,’ ‘bad mood,’ ‘fear,’ ‘tense,’ ‘helpless,’ ‘lonely,’ and ‘heavy.’ These feelings were often perceived as ‘normal’ within their communities:

They can't realize they have issues. For them it's normal live with fears, struggling, tense. You know you don't have a choice.

(CVC 27, Workshop 1)

#### 3.1.2 Displacement, Exclusion and Desire to Belong

Many participants described profound losses—of place, identity, and social status—alongside feelings of dislocation. Some spoke about leaving behind loved ones and family homes. Others mourned the loss of cultural places and artefacts that anchored their sense of self. For many, creating a ‘home’ was seen as essential for psychological safety:

As refugees (we) have to grapple with loss of identity, loss of place so there is a huge importance for creating a feeling of belonging and home here.

(CVC 16, Workshop 1)

Despite wanting to belong, participants often described exclusion from wider English society. Everyday cultural practices were sometimes misinterpreted as inappropriate or suspicious in the host context, reinforcing feelings of vulnerability and otherness. One example involved Afghan men being forced to move from where they regularly gathered after complaints to the local council:

Started to use this space as a place to meet, gather and wind down whilst watching their children play. For them it was very normal, but it looked wrong to others and there were complaints about it…a cultural practice in a space where it was deemed inappropriate, but it was important for them to be able to continue with their practice.

(CVC 16, Workshop 1)

Some participants mentioned heightened awareness of racial discrimination because of recent anti‐immigration protests and racial attacks across English towns and cities. Witnessing these events had intensified anxiety and safety concerns about accessing public spaces, particularly among women:

…where they think there won't be any racism or Islamophobia, especially, you know, with the riots, the Southport [where stabbing of girls by an ethnic minority teenager occurred] riots that started. Do you know there were so many women who used to go to the park together, mums after dropping children off to school, the mums would get together and go to parks. They completely stopped that.

(CVC 3, Workshop 3)

These experiences appeared to strengthen some participants' reliance on their own communities for safety and support. Others described withdrawing into isolation as a coping mechanism:

We miss in London community cohesion, mercy between individuals, sessions between neighbours. Everyone taking anything and run away. No trust. Very weak connections. When I first came here, I asked why are you doing this? They said, you will understand. Everyone will just take what they can get, run. Now I draw a line around me, no one enter until they are where I am.

(CVC 17, Workshop 1)

#### 3.1.3 Taboo, Shame and Silence

Mental distress was widely perceived as stigmatised within their communities, associated with weakness, failure and family dishonour. This seemed to discourage disclosure even within close‐knit families or communities. The social costs of gossip, blame, or exclusion being perceived as greater than any potential benefits of help‐seeking. Silence became a protective, but isolating response:

There are so many individuals in the community suffering silently because it's a taboo, again a taboo, so hide these situations from your families.

(CVC 6, Workshop 1)

A few participants, however, identified community‐based spaces, particularly with faith or outreach groups, as safe environments for discussing mental distress without stigma and judgement:

They have had members or people who have, like, who are quite a bit suicidal. And, you know, they felt being loved and being able to talk to someone, being able to open up, because you are there to serve the community, to actively listen to them. So, that can open doors for people to talk freely about what they are going through.

(CVC 15, Workshop 3)

### 3.2 Theme Two: Unknown Spaces and Unfriendly Environments

Limited awareness of CGCAs and their mental health benefits prevented many participants benefiting from the wellbeing opportunities they offered. Safety concerns, physical and emotional, also influenced engagement. For many, racialised fear, inadequate infrastructure, and restricted transport options combined to limit use of what should be open community spaces.

#### 3.2.1 Limited Awareness and Understanding of CGCAs

Participants frequently highlighted limited awareness of local CGCAs, unclear access requirements and uncertainty about costs. Available information was often judged inaccessible, fragmented, or not culturally or linguistically appropriate. The need to increase awareness of CGCAs through community‐targeted communications using trusted channels was often emphasised. While some participants valued digital and social media, others reported digital exclusion due to limited devices, skills or confidence. Traditional methods such as printed leaflets and posters in health centres, places of worship and community hubs, were viewed by many as essential for reaching those unconnected to online networks.

Several participants noted that community members were often unaware of the mental health benefits of engaging with CGCAs. They viewed community leaders and peer advocates as influential in building trust and sharing this knowledge, especially among older adults and recent migrants:

What I've noticed within my community…is that awareness is not quite there, the link between how beneficial open spaces could be…And I think when you engage with people and tell them that is what's available out there for you, and I think it needs more of that.

(CVC 6, Workshop 3)

For some, participating in the study deepened their understanding of how nature and culture support wellbeing:

One of the biggest lessons I've learned is how much nature can heal and find balance during tough times.

(CVC 5, Letter)

#### 3.2.2 Insecure and Unsafe About Accessing Spaces

Safety concerns were a commonly mentioned challenge to accessing CGCAs. Participants described racial stigmatisation, local violence, and discrimination as shaping fears about using public transport or walking to local CGCAs. Many viewed their neighbourhoods as physically unsafe or socially intimidating due to poor lighting, vandalism, and visible street crime:

There's lots of nitties [people dependent on drugs] in the area asking for money to buy drugs and being a public nuisance, making people feel uncomfortable and unsafe.

(CVC 22, Community member story)

Community transport services to CGCAs were valued for their safety and reliability. However, limited availability restricted who could benefit, particularly older adults and those with mobility challenges.

Uncertainty about staff support within CGCAs heightened feelings of vulnerability, especially for people with language difficulties or limited confidence in unfamiliar settings. Some participants expressed broader safety fears about public spaces including unleashed dogs or being stopped and detained by police.

### 3.3 Theme Three: Creating Spaces of Safety and Belonging

Participants frequently emphasised how spaces made them ‘feel.’ Physical access did not assure a sense of belonging. Emotional, social, and cultural dynamics shaped whether CGCAs made participants feel welcome, safe and included. Subtle cultural cues, social norms, and absence of visible inclusion within CGCAs often reproduced exclusion, reminding participants of who does and does not belong. Community‐led activities were seen as key to transforming CGCAs into inclusive and welcoming spaces.

#### 3.3.1 Spaces as Sanctuary

Many participants valued cultural and green spaces as sanctuary from everyday stresses. Some spoke about the benefits of peace, reflection, and ‘me‐time,’ others preferred socialising in places of calm. These environments could often evoke memories of childhood, homeland, and sensory familiarity, creating an emotional bridge between past and present, loss and restoration:

I remember how tranquil it was surrounded with trees, grass, and also at the same time how I felt life was so small and worthless…I felt so weak and lonely especially with dark and cloudy days. Trees, plants and soils bring the childhood I had back in [country migrated from] and I try to remember my childhood by finding nature and doing so, it brings the happiness, sadness and all the joys I had.

(CVC 7, Workshop 1)

#### 3.3.2 Cultural Dissonance and Exclusion

Despite their value, participants often felt excluded from CGCAs. Several described being alienated by the social and cultural behaviour of ethnic majority communities, especially in green spaces. Actions such as littering, uncontrolled dogs, and disregard for nature conflicted with participants' expectations of communal care:

The local park is not well kept, and many users bring dogs without lead and allowed to stay which makes me feel uncomfortable.

(CVC 15, Community member story)

Unfamiliar social norms, such as public sunbathing, also created cultural dissonance and a sense that certain spaces were ‘not for them.’ One participant, however, recounted a moment that reshaped their perception when they observed others from their community using a park for shared meals:

People are taking sun bath, like taking a suncream and they will, half naked, they will take a sun bath, mostly they are doing like that. But that time I saw [name] family, they had a lunch in park. And it looks like they were eating there…So, I after that, I told to my sister, one day we also going like, it's like a picnic.

(CVC 24, Workshop 3)

Safety and racialised belonging were also widespread concerns. Spaces perceived as dominated by ethnic majority groups or associated with violence were often avoided, especially after dark. Parents, in particular, voiced fears for their children's safety:

I think especially in the place that we live we can admit there are loads of stabbings that always happen in the green spaces, so no parent wants to send the child up by themselves to that green space.

(CVC 23, Workshop 3)

In addition to these barriers, participants lamented the absence of CGCAs that reflected their cultural heritage. A lack of events, activities, or visual representation of their communities reinforced the perception that these spaces were not ‘theirs’:

Some talked about feeling like there's nowhere truly theirs, where they can express their culture, creativity, or just take a moment to breathe… Imagine if we had more spaces where everyone felt welcome, more events that brought us together, more ways to celebrate who we are and where we're from.

(CVC 19 Letter)

#### 3.3.3 Navigating Unknown Rules and Institutional Mistrust

Uncertainty about entitlement to use certain CGCAs as well as the rules and expected behaviour were recurrent in participants' narratives. Many expressed worries about unintentionally doing the wrong thing and there being negative consequences. Some described needing reassurance that their presence was legitimate:

Very important thing for me to act with me with polite way. In London there is lack of community and lack of mutual respect.

(CVC 17, Workshop 1)

Institutional mistrust reinforced these concerns. Several participants described obscure council processes and perceived unfairness when engaging with public services:

Football group asked council if they can use derelict space but were turned down—lack of transparency about rules and regulations.

(CVC 3, Workshop 1)

#### 3.3.4 Community Makes Spaces Inclusive, Welcoming and Safe

Many participants highlighted the transformative impact of community‐led initiatives that could make CGCAs welcoming, safe and inclusive environments. Sharing cultural food, practices, traditions, and languages through activities such as community picnics or barbecues in local parks, fostered familiarity, cultural connection and belonging:

[Park barbecue]…I joined a group of aunties busy exchanging recipes and chatting about everything from food to family. Uncles shared stories, drawing laughter from the crowd, while even strangers found themselves sitting together and connecting over plates of food. There was a wonderful feeling of unity, knowing you were surrounded by people who understood your culture and traditions.

(CVC 5, Community member story)

Structured community‐run initiatives, such gardening groups, support groups and activity centres were also perceived to reshape spaces as inclusive and welcoming:

Within the centre you can understand where people are coming from without labelling them. Also, can have culturally relevant food, when eating together can sit side by side, often easier for people to talk that way.

(CVC 11, Workshop 1)

Several participants mentioned the restorative benefits of faith spaces including churches, temples and mosques that facilitated community connection:

It was a spiritual festival and a place where people could talk about their ideas of faith and feeling more connected. And feeling very peaceful and happy. There was a lot of singing and playing games and praying together and also food came into it, sharing a meal together.

(CVC 25, Workshop 2)

For some participants, visiting a museum where cultural artefacts from their community were displayed or seeing their cultural history represented in exhibitions evoked pride and a sense of respect. Cultural associations triggered by sensory experiences, such as community gardening, were said to foster belonging through shared memory:

We started some gardening project, which, you know, you see them kind of planting something…So, we've got people from different communities who say, “oh, yeah, you know, it brings back memories”.

(CVC 11, Workshop 3)

Acts of community resilience designed to make CGCAs feel safer were frequently mentioned, especially community members visiting in groups, including women‐only groups:

I think why those of ethnic minority communities they go with numbers is because of that discrimination. Because if you're by yourself you could be an easy target.

(CVC 18, Workshop 3)

They [Afghan women] formed a little walking group with themselves because they live near [name] Park. And this is something they did themselves…a solution to an issue.

(CVC 16, Workshop 3)

However, one participant cautioned that single‐community activities might unintentionally limit wider social connections:

It was clear that people were not connected outside of our small community and struggled to do so.

(CVC 10, Workshop 4)

#### 3.3.5 Community Facilitation and Peer Support

A few participants described using their community knowledge and trusted roles to encourage others to engage with CGCAs for mental wellbeing. Acting as community facilitators, they signposted isolated people experiencing distress, loneliness or bereavement to CGCAs with culturally relevant community activities that fostered companionship and support:

She's alone at home. And it's difficult. One of the things she raised, she's like, where can I get some cultural food? You know, that was one of the things. Where can I go to make some new friends?… So, I've signposted her to [name] luncheon club.

(CVC 5, Workshop 1)

Others recounted taking on a mediator role, facilitating apprehensive community members access to CGCAs by linking them with peers already engaged with activities in these spaces. This informal peer mentoring appeared to alleviate fear, build confidence, and normalise participation:

She heard about the numerous activities we organise so wanted the physical and mental stimulation, but didn't feel brave enough to attend…knowing someone else who attends made a huge difference so was not intimidated in any way.

(CVC 11, Community member story)

## 4 Discussion

This community‐based study used qualitative lived experience storytelling to examine the cultural drivers and mechanisms of inequalities hindering the engagement of underserved communities with CGCAs, and how these might be addressed to improve mental health. Applying Lamont et al.'s theoretical framework [46] generated new insights into how material, place‐based and symbolic inequalities restrict engagement and shape mental health disparities. The study also extends existing evidence by showing how symbolic and place‐based inequalities can be reduced when community‐led initiatives build trust and challenge symbolic boundaries. These findings are summarised in a conceptual model informed by the theoretical framework (Figure 1).

**Figure 1:**
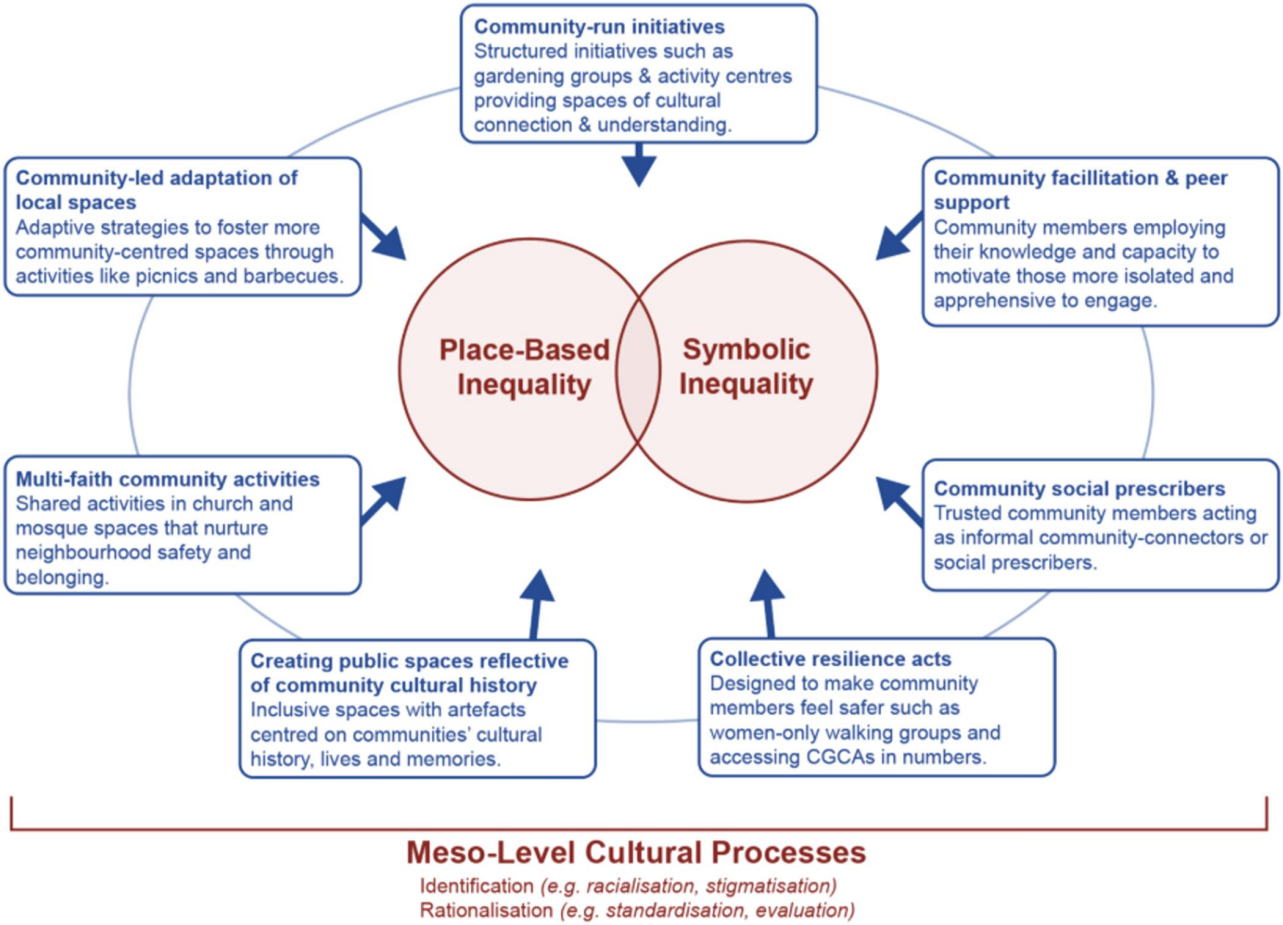
Reducing place‐based and symbolic inequalities through community‐led initiatives that support mental wellbeing.

Our study identified that the engagement of underserved communities with CGCAs is a dynamic, negotiated process shaped by cultural processes that influence the type, intensity, and persistence of inequalities. Participants frequently described experiences of stigmatisation and discrimination, including negative reactions to their cultural practices and anti‐immigration sentiment. These experiences, known to have detrimental effect on mental health [54], contributed to what Lamont et al. refer to as ‘symbolic boundaries that create the conditions for social boundaries’ [46], making some CGCAs feel unsafe and unwelcoming and limiting engagement with spaces that could support wellbeing. Social stigma surrounding mental illness within their communities [55], also constrained shared community discussion, understandings, and wellbeing support behaviours.

Study findings revealed limited awareness of local CGCAs and their mental wellbeing benefits, reinforcing symbolic inequalities. Even when CGCAs were available locally and known about, experiences of stigmatisation and discrimination in deprived neighbourhoods shaped perceptions of risk and discouraged engagement. Inadequate neighbourhood infrastructure often exacerbated these place‐based inequalities. Safety concerns—physical, psychological and social—dominated accounts of public transport, walking locally, and navigating CGCAs with limited English, reinforcing the material inequalities faced by underserved communities.

Belonging encompasses feeling safe, recognised, connected and secure. While members of dominant communities can often take this for granted without effort needed to fit in [56], our participants often described feeling out of place in their neighbourhood. Experiences of racial discrimination, violence and lack of diversity in CGCAs seemed to intensify feelings of exclusion and marginalisation, strengthening symbolic inequalities. Shared community narratives of certain CGCAs as hostile and unsafe discouraged use and heightened place‐based inequalities.

Some participants mentioned shunning spaces associated with unfamiliar or culturally conflicting practices, such as sunbathing, reinforcing social boundaries and symbolic inequalities. Language barriers conveyed to some that they were uninvited [57]. Uncertainty about unspoken ‘rules of the game’ [58], also caused concern, reflecting deeper institutional mistrust. These experiences align with Lamont et al.'s contention that in racialised societies, institutional rules shaped by historical inequities rarely operate in fair and equally accessible ways [46].

Lamont et al. nevertheless argued that cultural processes shaping inequalities are open‐ended and can be influenced by individual and community action [46]. Our findings support this view, showing that community‐led initiatives and agency helped mitigate symbolic and place‐based inequalities by bridging social boundaries around CGCAs (Figure 1). Community organisations reframed cultural belonging through activities rooted in shared cultural traditions, such as gardening groups, cookery clubs, arts and crafts, and inter‐faith events. Alongside acts of community solidarity, such as women‐only walking groups, these activities demonstrated collective resilience [59] and strengthened engagement with CGCAs. This accords with evidence that place‐identity is not fixed and can be a collective construction that fosters belonging and inclusion [60]. Study findings also highlighted the value of community members as informal community‐connectors or social prescribers [61], facilitating access to CGCAs through relational trust.

These community‐led initiatives demonstrated communitarian elements, meeting shared needs through mutual support that fostered engagement and collective wellbeing [62]. Without this community capacity to promote inclusion [63], CGCAs would struggle to function as genuine ‘community’ assets for underserved communities that can support their mental health.

Our findings have policy and practice implications. They show that increasing awareness and availability of CGCAs will not improve mental health in underserved communities if symbolic, place‐based and material inequalities that limit engagement remain unaddressed. Efforts to increase engagement should prioritise environments where underserved communities feel welcomed, represented and safe. Supporting culturally relevant activities delivered by local community organisations in cultural and green spaces can help bridge symbolic boundaries, strengthen belonging, and reduce place‐based and symbolic inequalities. To make CGCAs genuinely inclusive, investment in community capacity, transport, and neighbourhood safety is also needed to tackle material inequalities. More broadly, recognising CGCAs as part of neighbourhood mental ill‐health prevention infrastructure, supported through partnerships across health, local authority, and community and voluntary organisations, aligns with current policy ambitions to strengthen preventative care and reduce health inequalities [64].

### 4.1 Strengths and Limitations

The involvement of five community organisation leads as study co‐investigators facilitated recruitment of volunteer participants from diverse underserved communities and centring of the community voice. The lived experience storytelling design accorded with the custom of community members to tell stories to share knowledge and understanding. Positive feedback following the workshops was related to a sense of community empowerment and learning. Although considered rewarding, the collaborative process was also reported as emotionally demanding, highlighting a need for ongoing attention to support and safeguarding issues.

While CVCs were instrumental in communicating participants' stories, a limitation is that it was not possible to ascertain the extent to which narratives fully captured or potentially altered the essence of individual experiences. However, the stories offered a collective and authentic reflection of lived experience with valuable insights across diverse storytellers. Finally, use of qualitative methods may be considered a limitation, however, we have generalised our findings at a theoretical level.

## 5 Conclusion

Our study provides practical insights for policymakers seeking to reduce mental health inequities by increasing the engagement of underserved communities with CGCAs. Our findings showed that engagement is not only about access, but a negotiated process shaped by symbolic, place‐based and material inequalities. Stigma, discrimination, and exclusion continue to undermine community members' sense of belonging. Nevertheless, these inequalities are not fixed. Community‐led activities grounded in trust, cultural resonance and collective action can transform exclusionary environments into inclusive and welcoming ones to support mental health.

## Author Contributions

**Francesca Taylor:** writing – original draft, writing – review and editing, data curation, formal analysis, methodology. **Tushna Vandrevala:** funding acquisition, writing – review and editing, conceptualization, methodology, formal analysis, supervision. **Silke Zschomler:** writing – review and editing, data curation, formal analysis. **Meg Jensen:** funding acquisition, writing – review and editing, conceptualization, methodology, project administration, investigation. **Ima Miah:** funding acquisition, writing – review and editing, conceptualization, validation, resources, methodology. **Catherine Dawkins:** conceptualization, funding acquisition, methodology, validation, writing – review and editing. **Eneida Capaldi:** writing – review and editing, resources, validation. **Anna Bosher:** writing – review and editing, resources, validation. **Louise Bloom:** writing – review and editing, resources, validation. **Maria Chatzichristodoulou:** conceptualization, funding acquisition, writing – review and editing, methodology, supervision.

## Conflicts of Interest

The authors declare no conflicts of interest.

## Supporting information


Supporting File

## Data Availability

The data that support the findings of this study are available on request from the corresponding author. The data are not publicly available due to privacy or ethical restrictions.
