# Supplementary material for: Understanding Inequalities Shaping Engagement With Cultural and Green Assets to Improve Mental Health for Underserved Communities: A Qualitative Study
Source: Health Expect. 2026 Oct 8;29(5):e70912. doi: 10.1111/hex.70912 (PMC13648020; doi:10.1111/hex.70912)
Supplement: Supplementary file 1 [file HEX-29-e70912-s001.docx]

| **Workshop** | **Storytelling prompts** |
| --- | --- |
| **1** | You can either draw or write any words you like in any language you like. What are things that you think are important to your life and your world and your community?  Think of a recent challenge you faced related to mental health - yours or someone else's, a new or continuing, serious or minor worry or concern. How did you overcome it?  Think of a space or place that you spent time during this mental health challenge or another challenge. What made you want to go to that place?  What comes to mind when you think of the word ‘Nature’? Now what comes to mind when you think of the word ‘Culture’? Then the word ‘Mood’? And the last word ‘Health’? |
| **2** | Think about one time that the air or plants or trees or green life became important to you. What do you remember about that time? Why were the air or plants or trees or green life important to you at that time?  Think about a place and describe the sights, sounds, and smells? Where is this place and what are you doing? What did you come to this place hoping for or expecting? What does it feel like to be in this place, describe your feelings? |
| **3** | What has been your experience of using green and cultural spaces that are available in your community? These include things like local parks, churches, museums, theatres, garden centres, community centres. How do those experiences affect your personal life?  What have you picked up from your communities about green and cultural places, and thoughts and feelings to do with these places or spaces?  Imagine now looking seeing out of a window at your present-day life. What do you see and hear? Is there anything you would like to change in that image that you have? |
| **4** | Think about the stories you have collected from people in your community. What, if anything, was positive about this for you? What, if anything, was negative? What future opportunities for change and improvement in your community did you learn about through collecting stories?  Which three places or spaces of those you heard about were most comfortable, happy or relaxed to be in? Which three places or spaces you heard about were less comfortable to be in or could not easily be entered? What are three words you heard to describe the feeling of places or spaces that support or help people most?  What are the key green and cultural places and spaces in your community? Why do you and others use these places and spaces? How do you and others use them and why? |

# **Supplement A** Examples of storytelling prompts for CVC workshops
